# The sFlt-1/PlGF Ratio at 12, 24, and 32 Weeks Gestation in Twin Pregnancies as a Predictor of Late Preterm Birth and Perinatal Event Secondary to Prematurity

**DOI:** 10.3390/jcm13092699

**Published:** 2024-05-04

**Authors:** Elena Satorres-Pérez, Alicia Martínez-Varea, Blanca Novillo-Del Álamo, José Morales-Roselló, Vicente Diago-Almela

**Affiliations:** 1 Department of Obstetrics and Gynecology, La Fe University and Polytechnic Hospital, Avenida Fernando Abril Martorell 106, 46026 Valencia, Spain; elenasatorres@gmail.com (E.S.-P.); bnalamo@gmail.com (B.N.-D.Á.); cm@comv.es (J.M.-R.); diago_vicalm@gva.es (V.D.-A.); 2 Department of Pediatrics, Obstetrics and Gynecology, Faculty of Medicine, University of Valencia, 46010 Valencia, Spain; 3 Department of Medicine, CEU Cardenal Herrera University, 12006 Castellón de la Plana, Spain; 4 Faculty of Health Sciences, Universidad Internacional de Valencia, 46002 Valencia, Spain

**Keywords:** twin pregnancy, sFlt-1/PlGF ratio, preterm birth, adverse perinatal outcomes, prematurity

## Abstract

**Background:** Preterm birth impacts 60% of twin pregnancies, with the subsequent risk of complications in both newborns secondary to the immaturity of organs. This study aims to assess the utility of the sFlt-1/PlGF ratio throughout pregnancy in predicting late preterm birth and adverse perinatal outcomes related to prematurity in twin pregnancies. **Methods:** This is a prospective cohort study developed at a tertiary hospital. All pregnant women with a twin pregnancy who signed the informed consent were included. The sFlt-1/PlGF ratio was measured at 12, 24, and 32 weeks’ gestation. **Results:** Seventy patients were included, from which 54.3% suffered late preterm birth. Results revealed a significant difference in sFlt-1/PlGF ratio at week 32 between term and preterm groups, with a one-unit increase associated with a 1.11-fold increase in the probability of preterm birth. The sFlt-1/PlGF ratio at week 32 alone presented considerable predictive capacities (sensitivity of 71%, specificity of 72%, a PPV of 75%, and an NPV of 68%. Similarly, at week 24, a one-unit increase in sFlt-1/PlGF ratio was associated with a 1.24-fold increase in the probability of adverse perinatal events due to prematurity. Combining parity, maternal age, conception method, BMI, and chorionicity, the model yielded better predictive capacities (sensitivity of 82%, specificity of 80%, PPV of 58%, NPV of 93%). **Conclusions:** The potential of the sFlt-1/PlGF ratio as a predictive tool for preterm birth and adverse perinatal outcomes secondary to prematurity in twin pregnancies is underscored.

## 1. Introduction

Preterm birth, defined as delivery before 37 weeks of gestation, impacts 5–18% of pregnancies [1]. It stands as the primary cause of neonatal mortality and ranks second among causes of childhood death before the age of 5, as neonates born preterm face an elevated risk of short-term complications secondary to the immaturity of organs [2]. Spontaneous preterm labor accounts for 70% of preterm births [3], and current understanding explains its initiation by multiple mechanisms, including infection or inflammation, uteroplacental ischemia or hemorrhage, uterine overdistension, stress, and other immunologically mediated processes [4,5,6,7,8,9,10,11].

Multiple pregnancies, although representing only 2–3% of infants, pose a substantial risk of preterm delivery and contribute to 15–20% of all preterm births. Approximately 60% of twins are born preterm, as almost 40% of twins experience spontaneous labor or preterm premature rupture of membranes (PPROM) before 37 weeks gestation, while others undergo indicated preterm delivery due to conditions such as pre-eclampsia or other maternal or fetal disorders [12,13,14]. Nevertheless, uterine overdistension is believed to be the major mechanism for the increased rate of spontaneous preterm births [15]. Newborns from multiple pregnancies account for approximately 15% of perinatal mortality [16].

In conditions such as hypoxia or placental stress, anti-angiogenic factors like soluble fms-like tyrosine kinase-1 (sFlt-1) are released into the maternal circulation, which subsequently reduces the availability of pro-angiogenic factors, including placental growth factor (PlGF) [17,18,19]. The sFlt-1/PlGF ratio as an indicator of placental dysfunction has been widely studied over the last decade [20]. Thus, numerous national and international societies have already included its determination in their protocols [21,22]. The anti-angiogenic factor sFlt-1 is a soluble protein that induces peripheral vasoconstriction, aiming to recruit additional blood volume and, secondarily, reduce ischemia [23]. Given that sFlt-1 binds to the domains of two pro-angiogenic proteins, PlGF and vascular endothelial growth factor (VEGF), it naturally decreases free PlGF levels [24]. A high ratio of placental biomarkers in the first trimester of pregnancy and an increased risk of spontaneous preterm birth and other pregnancy complications have previously been established [25,26], and its elevation in the third trimester is believed to be associated with placental hypoxia-related stress and an increased risk of complications [17]. Nevertheless, these studies have primarily assessed singletons, though evidence has proved that twin pregnancies have higher sFlt-1/PlGF ratios both in uncomplicated and complicated cases [27,28]. Therefore, this analysis aimed to assess whether the ratio of soluble fms-like tyrosine kinase 1 (sFlt-1) to placental growth factor (PlGF, the sFlt-1/PlGF ratio) measured at weeks 12, 24, or 32 could predict the risk of spontaneous preterm birth in twin pregnancies.

## 2. Materials and Methods

### 2.1. Study Design

This was a prospective cohort study among both mono and dichorionic twin pregnancies that were followed up at the University and Polytechnic Hospital La Fe, Valencia, Spain, from February 2021 to September 2023. The evolution of the sFlt-1/PlGF ratio during pregnancy was monitored at 12, 24, and 32 weeks of gestation using Elecsys^®^ immunoassay sFlt-1/PlGF ratio (Roche Diagnostics, Basel, Switzerland) in an outpatient setting. Serum samples were immediately analyzed (<6 h) after the collection. Gestational age, chorionicity, and amnionicity were defined by ultrasound on the first-trimester evaluation (11^+0^ to 13^+6^ weeks of pregnancy).

### 2.2. Inclusion and Exclusion Criteria

All women over 18 years old, pregnant with a twin pregnancy, with no fetal abnormalities, and who provided signed informed consent were included. Exclusion criteria included triplets or high-order pregnancies, and termination of pregnancy prior to complete sample collection, whether through birth or stillbirth. Additionally, as our protocol included assessments of the sFlt-1/PlGF ratio at weeks 12, 24, and 32 of pregnancy, any preterm births occurring before 32 weeks, as well as participants who voluntarily withdrew from the study before completion, were excluded from the analysis.

### 2.3. Outcome Data

The primary outcome was the event of preterm birth, defined by delivery between 32+0 weeks and 36+6 weeks. The comparison group was determined by a delivery at term, which occurred after 37+0 weeks. The secondary outcome evaluated the presence of an adverse perinatal event secondary to prematurity, defined by the existence of at least one of the following syndromes or conditions: surfactant deficiency disease, bradycardic apnea syndrome, non-immune jaundice, meningitis, hypoglycemia, newborn metabolic bone disease, respiratory distress, hyaline membrane disease, necrotizing enterocolitis, and meconial obstruction. These conditions were selected by a neonatologist as strictly related to preterm birth among all diagnoses collected from the included newborns. Furthermore, when found to be significant, the strength of the sFlt-1/PlGF ratio and each outcome correlation was evaluated. It was also assessed if the evolution between each sFlt-1/PlGF ratio determination was related to the studied events.

Data on patients concerning pregnancy and delivery were collected from their digital clinical history: maternal age, body mass index, parity, toxic habits, chronic diseases, chronic treatments, conception method, weight gain along the pregnancy, diagnosis of gestational diabetes or placental dysfunction related syndromes, mean uterine artery pulsatility index at week 12, gestational age at birth, mode and onset of delivery, neonatal outcome (weight, Apgar test, arterial and venous pH, admission to neonatal unit). All these parameters were compared between patients developing preterm birth and those who did not. Registered outcomes were collected through online clinical history and personal interviews if patients gave birth in a different hospital.

### 2.4. Statistical Analysis

Statistical analysis was performed using R. Descriptive analysis of all variables was performed, stratifying by patients showing preterm birth or at term. The Chi-squared statistical test assessed the statistical relationship between each categorical variable and the occurrence of preterm birth and adverse perinatal outcomes. For continuous variables, where the assumption of normality in data distribution was not met, we employed a non-parametric Mann–Whitney test to evaluate the statistical relationship between each continuous variable and the incidence of preterm birth as well as adverse perinatal outcomes. A mix-model ANOVA with factors time (week 12, week 24, and week 32) and group (Pre-term, at Term) was applied to test differences in the ratio across time and groups. Post hoc Tukey tests corrected by multiple comparisons were applied.

Multivariate Logistic regression models were used to test the relationship between the different variables and the occurrence of preterm birth and adverse perinatal outcomes. For each model, the Odds Ratio (OR) with the confidence interval at 95% (95% CI) was associated with each variable, and its statistical significance was reported. The predictive capacities of each of these models related to the occurrence of each event were also determined. In each analysis, accuracy, sensitivity, specificity, as well as the AUC-ROC curve were reported. In all cases, statistical significance was set at *p* < 0.05.

### 2.5. Ethics

The study was approved by the Ethics Committee of the Health Research Institute Hospital La Fe (IIS La Fe). All pregnant women signed the informed consent form before participating in the study.

## 3. Results

A total of 70 patients with a twin pregnancy were included in our study, both monochorionic (14.29%) and dichorionic (85.71%). In all these collected twin pregnancies, the sFlt-1/PlGF ratio was determined at 12, 24, and 32 weeks of gestation. Initially, 107 patients signed the informed consent document and accepted participation. Nonetheless, 29 voluntarily withdrew from the study for personal reasons, 7 gave birth before week 32, and 1 suffered stillbirth and therefore did not complete sample collection. Data regarding baseline characteristics, comparing pregnant women who had preterm birth with those who did, are displayed in Table 1.

From the final 70 included patients, 38 (54.3%) presented late preterm birth, while 32 (45.71%) reached week 37 of pregnancy. A significant relationship (Fisher test, *p* value = 0.0014) was found in the proportion of cases with preterm birth based on chorionicity. Out of the total dichorionic cases (*n* = 60), 46.7% experienced late preterm birth. Conversely, all 10 monochorionic cases exhibited late preterm birth (*n* = 100%). Additionally, significant differences (*p*-value < 0.05) were found between groups in both newborns’ birth weights based on the presence or absence of preterm birth.

In order to assess the relationship between placental dysfunction-related syndromes and preterm birth, fetal growth restriction (FGR) and preeclampsia diagnosis were collected. In our sample, 55.26% of the patients presenting preterm birth were not diagnosed with FGR or preeclampsia. The association between placental dysfunction and the birth at term or preterm was statistically significant (*p* = 0.004). The odds ratio was 5.52, with a 95% CI of [1.5, 25.94]

**Figure 1:**
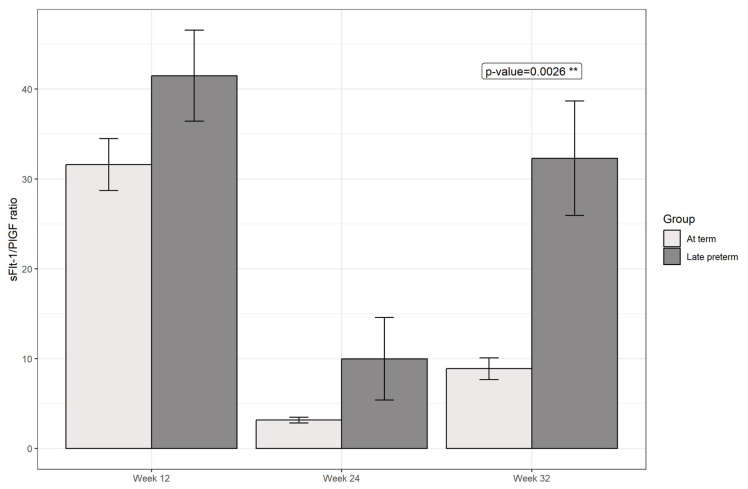
Evolution of hormonal ratio based on the presence of late preterm birth (No: light gray, Yes: dark gray). Error bars represent the standard error. ** Represents *p*-value <0.01.

A repeated-measures analysis of variance (ANOVA) with factors group (at term, preterm) and time (12, 24, 32 weeks) was conducted to test whether the ratio sFlt-1/PlGF levels across the three distinct time points differed between groups. By incorporating a random effect using an identification for each patient, the model accounted for individual variations among patients. Statistically significant differences were found between the observed sFlt-1/PlGF ratio at week 32 based on the presence of late preterm birth (*p* = 0.0026, corrected by the Tukey test for multiple comparisons), as seen in Figure 1 and Table 2.

**Figure 2:**
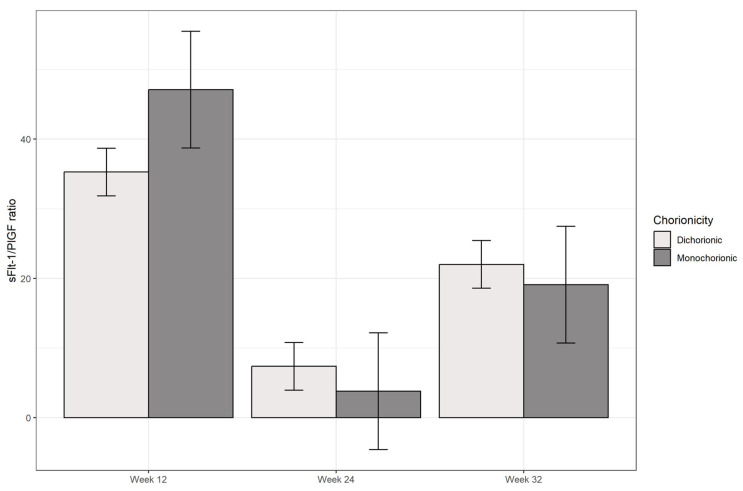
Evolution on sFlt-1/PlGF ratio in monochorionic vs. dichorionic pregnancies.

As seen in Figure 2 and Table 3, no statistically significant differences were found in the three determinations between monochorionic and dichorionic pregnancies.

**Figure 3:**
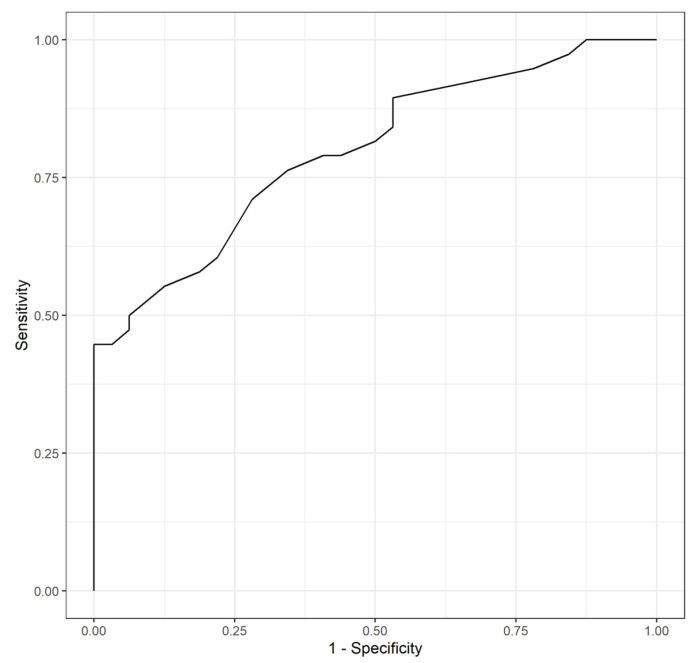
AUC- ROC curve for sFlt-1/PlGF determination at week 32 for the detection of patients with twin pregnancies who subsequently develop preterm birth.

After evaluating the relationship between the three determinations with the occurrence of preterm birth through a logistic regression model, it was found that the OR related with the sFlt-1/PlGF ratio at week 32 was statistically significant (coefficient = 0.107147; *p* = 0.00104), with an associated OR of 1.11 (1.05–1.20). The changes between all three measurements were also evaluated, and statistically significant differences were found in the evolution between weeks 24 and 32 (ascending levels of sFlt-1/PlGF), with a 1.1 OR (1.05–1.19; *p* = 0.005). This means that the increase of one unit of sFlt-1/PlGF ratio difference between week 24 and week 32 increased the probability of presenting a preterm birth with respect to not presenting this event by 1.1. Furthermore, it was evaluated if adding other data increased the predictive power of sFlt-1/PlGF alone concerning preterm birth. A logistic regression model combined the parity, maternal age, conception method, and BMI with the sFlt-1/PlGF ratio level at week 32. The model presented statistically significant results with respect to the null model (*p* = 0.0002) but did not statistically improve the predictive value of the sFlt-1/PlGF ratio alone (Likelihood ratio test, *p* > 0.05). Therefore, we proposed to use the most parsimonious model, including only the ratio alone instead of adding additional variables. As represented in Figure 3, an AUC-ROC curve was performed to analyze the predictive capacities of such a model. A ratio >12 at 32 weeks was related to a sensitivity of 71%, specificity of 72%, and an accuracy of 71%, with a positive predictive value (PPV) of 0.75 and a negative predictive value (NPV) of 0.68 for the identification of patients who develop preterm birth.

Regarding the occurrence of an adverse perinatal event, 19 women had children with one or more diagnoses secondary to prematurity. Data regarding perinatal outcomes is summarized in Table 1.

**Figure 4:**
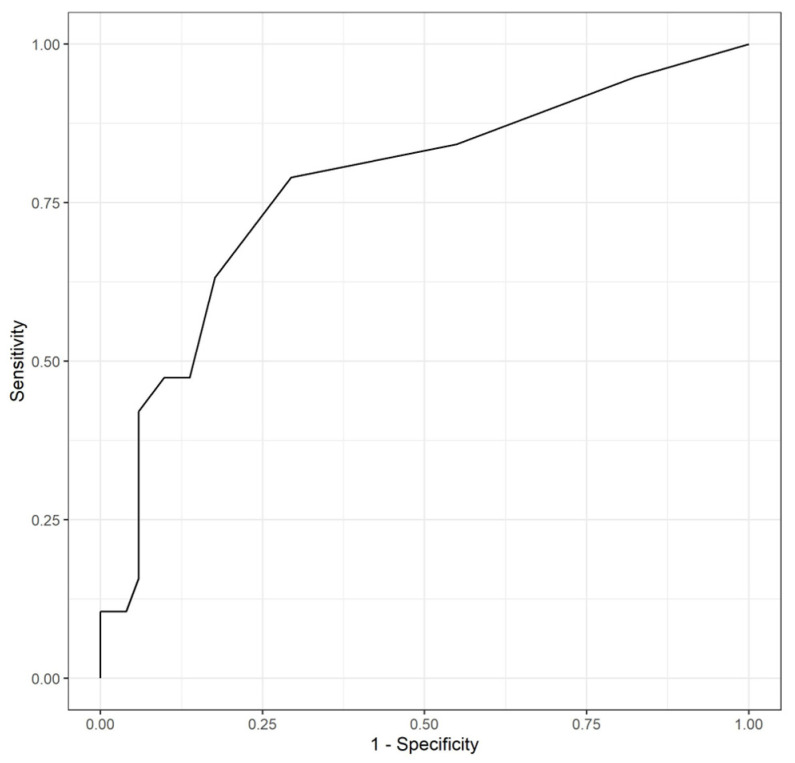
AUC-ROC curve for sFlt-1/PlGF determination at week 24 for the detection of patients with twin pregnancies who subsequently develop adverse perinatal events secondary to prematurity.

**Figure 5:**
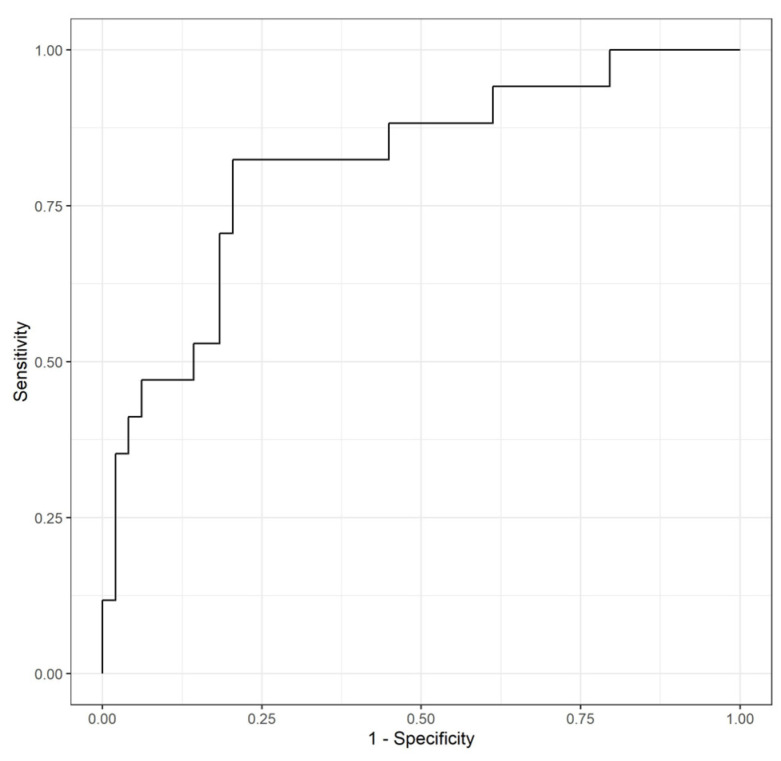
AUC- ROC curve for sFlt-1/PlGF determination at week 24, parity, maternal age, conception method, BMI, and chorionicity for detecting patients with twin pregnancies who subsequently develop adverse perinatal events secondary to prematurity.

When evaluating the relationship between the three determinations of sFlt-1/PlGF ratio with the occurrence of an adverse perinatal event through a logistic regression model, statistically significant differences were found for week 24, with an OR of 1.24 (1.11–1.40; *p* = 0.0002). Thus, the increase of one unit of the sFlt-1/PlGF ratio increased the probability of presenting an adverse perinatal event secondary to prematurity by 1.40. The evolutive changes between the three measurements of the sFlt-1/PlGF ratio at 12, 24, and 32 weeks gestation were also evaluated, and statistically significant differences were found in the change between week 24 and 32 (ascending levels of sFlt-1/PlGF), with a 1.04 OR (1.01–1.07; *p* = 0.0301). Furthermore, it was also evaluated if adding other data increased the predictive power of the sFlt-1/PlGF ratio alone. Thus, parity, maternal age, conception method, BMI, and chorionicity were combined with ratio level at week 24, after standardizing data due to differences in scales. The model presented statistically significant results with respect to the null model for ratio at week 24 and chorionicity. Furthermore, it statistically improved the predictive value of the sFlt-1/PlGF ratio alone (Likelihood ratio test, *p* = 0.04). In this model, ratio levels at week 24 presented an OR of 314 (25–5795; *p* = 0.00002). Thus, an increase of one standard deviation meant increasing the probability of presenting an adverse perinatal event by 314. As for chorionicity, an OR of 6.88 (1.57–33.04; *p* = 0.01) was found for monochorionic pregnancies. Additionally, as represented in Figure 4, an AUC-ROC curve was performed to analyze the predictive capacities of the model, including the sFlt-1/PlGF ratio, for week 24 only. A sFlt-1/PlGF ratio at week 24 > 4 weeks was related to a sensitivity of 79%, a specificity of 71%, an accuracy of 73%, with a PPV of 0.50 and a negative predictive value NPV of 0.90, were found. This AUC-ROC curve was subsequently compared to a second one, which included all additional data (Figure 5)*,* with higher predictive values: a sensitivity of 82%, a specificity of 80%, and an accuracy of 80%, with a PPV of 0.58 and a negative predictive value NPV of 0.93.

## 4. Discussion

The main finding of this study was that maternal serum levels of sFlt-1/PlGF ratio at week 32 in women with a twin pregnancy were strongly associated with preterm birth. Additionally, maternal serum levels of sFlt-1/PlGF ratio at week 24 in patients with a twin pregnancy, especially associated with additional pregnancy data (parity, maternal age, conception method, BMI, and chorionicity), were strongly associated with adverse neonatal events related to prematurity.

A significant difference in the sFlt-1/PlGF ratio at week 32 between at-term and preterm groups was revealed when evaluating gestational age at birth. An increase of one unit in such determination increased the probability of presenting preterm birth by 1.11, and a difference of one unit between the sFlt-1/PlGF ratio at weeks 24 and 32 also entailed an increase of such probability by 1.1. Also, it was revealed that adding other variables, such as parity, maternal age, conception method, BMI, and chorionicity, did not enhance its predictive power. Therefore, the most parsimonious model was found to be the one using sFlt-1/PlGF ratio at week 32 only, as incorporating the evolution of precedent determinations or other data did not significantly improve the predictive power and implied using a more complex model. The preferred model presented notable sensitivities and specificities (71 and 72%, respectively).

Conversely, regarding adverse perinatal outcomes, the association between the sFlt-1/PlGF ratio levels at week 24 and the occurrence of such events was found to be statistically significant. An increase of one unit in the sFlt-1/PlGF ratio at week 24 was related to an increase in the probability of developing an adverse perinatal event secondary to prematurity by 1.24. Similar findings were discovered when analyzing the evolution between all three determinations, as the increase of one unit on the difference between sFlt-1/PlGF ratio levels at weeks 24 and 32 implied an increase in the probability of presenting the event by 1.04. By combining parity, maternal age, conception method, BMI, and chorionicity, the predictive power of the sFlt-1/PlGF ratio alone was optimized (Likelihood ratio test, *p* = 0.04). In this model, an increase of one standard deviation meant raising the probability of presenting an adverse perinatal event by 314. Additionally, AUC-ROC curves were performed to analyze the predictive capacities of both models, with better numbers obtained by the model, including additional data (AUC of 77 vs. 82%, sensitivity of 79 vs. 82%; specificity of 71 vs. 80%, PPV of 50 vs. 58%; NPV of 90 vs. 93%). Therefore, when using sFlt-1/PlGF as a predictor of adverse perinatal events associated with prematurity, the most parsimonious model was found to be the one that included determination at week 24 plus the mentioned pregnancy characteristics.

Although the sFlt-1/PlGF ratio has been mainly studied and applied to detect placental dysfunction, in our analyzed sample of patients with a twin pregnancy, only 44.74% of the women presenting preterm birth had an established diagnosis related to placental dysfunction, such as preeclampsia or fetal growth restriction [29,30,31,32,33,34]. This leads to a 55.26% remaining patients with a twin pregnancy without a diagnosis related to placental dysfunction, such as FGR or preeclampsia, in which the alteration of placental biomarkers such as the sFlt-1/PlGF ratio could be due to alternative physiopathological mechanisms, as reported in previous studies [35,36,37]. It has been widely proposed that spontaneous preterm birth can be initiated secondary to other multiple conditions, including uterine ischemia, infection, or fetal stress [38,39,40]. However, the exact mechanisms underlying the onset of preterm birth in these cases remain undetermined.

The presented findings of this study support the potential utility of placental markers determined in maternal serum for predicting preterm birth secondary to any cause. These results are in accordance with those reported in a recent article, where an increase in the maternal serum level of sFlt-1/PlGF between 20 and 28 weeks was strongly associated with the subsequent risk of spontaneous preterm birth (50% for a 1 SD increase in the level of the ratio) [36]. According to such findings, an American group found that decreased levels of PlGF in the first two trimesters of pregnancy were associated with medically indicated preterm birth [26]. Previous studies have already proposed this relationship, with significant associations at 28 weeks of gestation or approximately 5–10 weeks prior to the diagnosis [37]. Along the same line, other maternal and placental biomarkers have also been assessed to predict preterm birth, but with contradictory results [41,42]. Nevertheless, these studies are mostly centered on single pregnancies. To the best of our knowledge, there is no published research assessing placental biomarkers for preterm birth prediction, specifically in twin pregnancies [34]. The development and incorporation of a potentially valuable tool for identifying high-risk patients might help optimize the decision-making for clinicians and improve the selection of patients benefiting from the application of invasive interventions, such as the use of tocolytic agents and corticosteroids for lung maturation. This could be especially useful in twin pregnancies, considered a high-risk group for preterm birth. Additionally, our findings suggest that placental serum biomarkers have a promising utility in identifying newborns at risk of developing an adverse perinatal outcome related to prematurity. This could benefit pediatricians and obstetric multidisciplinary teams in planning specific medical care for both newborns.

The main strength of this study is that, to the best of our knowledge, this is the first study to evaluate the role of placental biomarkers for preterm birth prediction and neonatal outcomes in twin pregnancies. Our results prove that the sFlt-1/PlGF ratio correlates with preterm birth and adverse perinatal outcomes in twin pregnancies. These associations underscore the potential impact of monitoring the sFlt-1/PlGF ratio in twins, as it could provide clinicians with valuable information for managing a condition with a considerable impact on perinatal and neonatal health. The most significant limitation relies on its restricted sample size, which influenced several variables, resulting in observable trends that did not reach statistical significance. The limited statistical power from the small sample size restricts the ability to make conclusive statements and underscores the need for caution in generalizing the findings. Plus, our sample only included patients with sFlt-1/PlGF ratio determination at week 32, which implied preterm births occurring before such week were not analyzed. Future research needs to utilize larger cohorts to validate and reinforce the identified trends, thereby improving the credibility of the study results.

## 5. Conclusions

The primary findings of this study suggest a strong association between maternal serum levels of the sFlt-1/PlGF ratio at week 32 and the incidence of preterm birth. Additionally, a notable correlation was observed at week 24, specifically concerning adverse neonatal outcomes related to prematurity. Differences in the sFlt-1/PlGF ratio at week 32 between term and preterm births were significant, with an increase in this ratio correlating with an increased risk of preterm birth. Nonetheless, incorporating additional variables such as parity, maternal age, conception method, BMI, and chorionicity did not enhance predictive power, suggesting that utilizing the sFlt-1/PlGF ratio alone at week 32 could be the most effective approach. Conversely, associations were found between sFlt-1/PlGF ratio levels at week 24 and adverse perinatal events, with higher predictive power when associated with other pregnancy data. While this study provides valuable insights into the utility of placental biomarkers for predicting preterm birth and adverse neonatal outcomes in twin pregnancies, further research with larger cohorts is required to validate these findings and enhance their reliability.

## Figures and Tables

**Table 1:** Baseline characteristics comparing women and newborns presenting preterm birth vs. birth at term. BMI = Body mass index; ART = assisted reproductive technology. Data are presented as median (IQR, Interquartile Range) for continuous variables or as *n* (%) for dichotomic variables.

Characteristics	At Term Birth (*n* = 32)	Preterm Birth (*n* = 38)	*p* Value
Age (years)	35.5 (32.8–37.2)	35.0 (31.2–38.8)	0.93
BMI	23.2 (21.2–24.8)	23.2 (20.4–25.4)	0.82
BMI > 35	4 (50)	4 (50)	1.00
Weight gain > 15 kg	11.5 (9.0–14.2)	11.5 (9.0–14.0)	0.88
Smoking habit	3 (42.86)	4 (57.14)	1.00
Nulliparous	22 (43.14)	29 (58.86)	0.48
Natural conception	12 (44.4)	15 (55.56)	0.73
Conception by ART	19 (48.72)	20 (51.28)	
Monochorionic	0 (0)	10 (100)	0.0014
Dichorionic	32 (53.33)	28 (46.67)	
Gestational Diabetes	4 (50)	4 (50)	1.00
Placental dysfunction	4 (12.5)	17 (44.74)	0.004
No placental dysfunction	21 (55.26)	28 (87.5	
Birth at weight 1st newborn (grams)	2565.0 (2420.0–2743.8)	2027.5 (1792.5–2387.5)	<0.0001
Birth at weight 2nd newborn (grams)	2432.5 (2257.5–2626.2)	2160.0 (1880.0–2450.0)	<0.0012
Apgar test at 5 min 1st newborn	10.0 (10.0–10.0)	10.0 (10.0–10.0)	0.16
Apgar test at 5 min 2nd newborn	10.0 (9.8–10.0)	10.0 (10.0–10.0)	0.73
Arterial pH 1st newborn	7.3 (0.1)	7.3 (0.1)	0.17
Venous pH 1st newborn	7.3 (0.04)	7.3 (0.1)	0.89

**Table 2:** Hormonal ratio levels along the three determinations, based on the presence of late preterm birth. Data are presented as mean and SD.

SFlt-1/PlGF Ratio	At Term Birth (*n* = 32)	Preterm Birth (*n* = 38)	*p* Value
Week 12	31.59 (2.9)	41.49 (5.07)	0.58
Week 24	3.16 (0.33)	9.98 (4.60)	0.87
Week 32	8.88 (1.22)	32.31 (6.37)	0.0026

**Table 3:** Hormonal ratio levels along the three determinations, based on chorionicity (monochorionic vs. dichorionic pregnancies). Data are presented as mean and SD.

SFlt-1/PlGF Ratio	Monochorionic (*n* = 10)	Dichorionich (*n* = 60)	*p* Value
Week 12	47.1 (8.78)	35.22 (3.28)	0.78
Week 24	3.8 (0.81)	7.37 (2.94)	0.99
Week 32	19.1 (4.64)	22.01 (4.32)	0.99

## Data Availability

The data in this study were obtained from the clinical program of La Fe University and Polytechnic Hospital. Further inquiries can be directed to the corresponding author.

## Abbreviations

ART	Assisted reproductive technology
AUC-ROC curve	Area Under the Curve–Receiver Operating Characteristic
BMI	Body mass index
CI	Confidence Interval
FGR	Fetal growth restriction
IQR	Interquartile range
NPV	negative predictive value
OR	Odds Ratio
PlGF	Placental growth factor
PPV	positive predictive value
PROM	Premature rupture of membranes
SD	standard deviation
sFlt-1	Soluble fms-like tyrosine kinase 1
